# IFN-Lambda 3 Mediates Antiviral Protection Against Porcine Epidemic Diarrhea Virus by Inducing a Distinct Antiviral Transcript Profile in Porcine Intestinal Epithelia

**DOI:** 10.3389/fimmu.2019.02394

**Published:** 2019-10-17

**Authors:** Liang Li, Mei Xue, Fang Fu, Lingdan Yin, Li Feng, Pinghuang Liu

**Affiliations:** State Key Laboratory of Veterinary Biotechnology, Harbin Veterinary Research Institute, Chinese Academy of Agricultural Sciences, Harbin, China

**Keywords:** interferon-lambda 3, interferon alpha, RNA-seq, enteroids, PEDV

## Abstract

Type III interferon-lambda (IFN-λ) plays a critical role against infection, particularly in mucosal infection in the respiratory and gastrointestinal tract. Our study and other previous studies have shown that porcine IFN-λ more efficiently curtails the infection of porcine epidemic diarrhea virus (PEDV) in the intestine epithelia than type I IFN, whereas IFN-λ3 exerts a more potent effect than IFN-λ1. However, the underlying mechanism remains elusive, and in particular, the transcriptional profile induced by IFN-λ3 has not been reported. Here, to resolve the mechanism responsible for the disparity between IFN-λ3 and type I IFN in anti-mucosal virus infection, we compared the transcription profiles induced by the two IFNs in porcine intestinal epithelial (IPEC-J2) cells by RNA-Seq. Our results showed that the pretreatment of IPEC-J2 cells with IFN-λ3 resulted in the differential expression of 983 genes. In contrast, IFN-α only modified the expression of 134 genes, and 110 of these genes were also observed in the response to IFN-λ3. A transcriptional enrichment analysis indicated that IFN-λ3 or IFN-α regulates multiple cellular processes and that IFN-λ3 activates more robust signaling pathways, particularly the antiviral Jak-STAT signaling pathway, than IFN-α. Furthermore, we verified the RNA-Seq results through an RT-qPCR analysis of IPEC-J2 cells and porcine enteroids. Moreover, transient expression of the porcine *rsad2* and *mx2* genes among the top 10 genes induced by IFN-λ3 significantly inhibited PEDV infection. Collectively, the data showed that IFN-λ3 induces a unique transcriptional profile that does not completely overlap with that induced by IFN-α and strongly elicits a set of genes responsible for the antiviral activity of IFN-λ3. These findings provide important knowledge regarding the elicited ISGs of type I and III IFNs in restricting porcine intestinal viral infection.

## Introduction

The surface epithelia of the mucosa are the major entry site of most pathogens in the host and serve as a first line of defense against invading pathogens. One of the most important antiviral cytokines in the host is interferons (IFNs), which perform key roles in inhibiting viral infection (1, 2). The IFN family is categorized into three different types: type I IFN (IFN-α/β), type II IFN (IFN-γ), and type III IFN (IFN-λ). Type II IFN, which is primarily produced by T cells and natural killer cells, exerts limited direct antiviral activity and plays a key role in modulating the host immune response (3), whereas type I IFNs (α/β) and the more recently discovered type III IFNs induce a strong antiviral state in responsive cells and play crucial roles in controlling viral infection (4–8). Although type I IFNs have generally been thought to be a key element against systemic infections, recent research has shown that IFN-λ plays a critical role in mucosal infections, such as enteric infection (9, 10). Unlike type I IFNs that are secreted by a wide range of different cell types upon stimulation, type III IFNs are primarily produced by epithelial cells, NK cells, and dendritic cells (DCs) (8, 11–13). IFN-λ acts primarily on the mucosal epithelium, which might result in fewer side effects compared with type I IFN treatment (8). These features make IFN-λ a potentially superior antiviral therapeutic candidate against local mucosal infection (7).

Although the receptors for type I and III IFNs are different, the binding of both type I and III IFNs to their corresponding receptors stimulates a Janus kinase (JAK)-signal transducer of transcription (STAT) pathway, and the stimulation of this pathways subsequently drives the transcription of IFN-stimulated genes (ISGs) and prompts cells toward an antiviral status (14). Consistent with the similarity of the induced signaling pathways, the spectrum of genes elicited by the two types of IFNs show a high overlap (2). However, recent studies have demonstrated that type III IFNs are critical non-redundant antiviral mediators of type I IFNs in the GI tract (2). To date, numerous studies in humans or mice have taken advantage of RNA-Seq or chip assays to show that IFN-λ and IFN-α elicit distinct downstream signaling events, even though many genes are induced by both type I and III IFNs (15, 16). Mice with type I IFN or III IFN receptor knockout experience more severe viral intestinal infections, but *Ifnl*^−/−^ mice show higher viral loads and more serious clinical symptoms than IFNAR^−/−^ mice (17, 18). Studies conducted by Pott et al. showed that intestinal epithelial cells exhibit stronger responses to IFN-λ compared with IFN-α/β *in vivo* (19, 20). A comprehensive understanding of the unique signaling profiles of type I and III IFNs has become increasingly important for understanding host–virus interactions and the development of IFN-λ therapeutics. However, thus far, no direct comparative analyses of the transcriptional profiles induced by porcine type I vs. type III IFNs in swine intestinal epithelia have been performed.

The piglet diarrhea caused by enteric coronavirus porcine epidemic diarrhea virus (PEDV) is a highly contagious disease characterized by watery diarrhea, dehydration, and causes up to 100% mortality in neonatal piglets. We and other research groups previously reported that porcine IFN-λ results in better suppression against PEDV infection compared with IFN-α and that IFN-λ3 more efficiently inhibits PEDV than IFN-λ1 (21–23). However, the mechanisms underlying the difference among IFN-λ1, IFN-λ3, and IFN-α in inhibiting enteric coronavirus remain less clear. Previous studies have largely focused on the gene profiles induced by human or mouse IFN-λ1 and IFN-α, but the IFN-λ3- and IFN-α-elicited genes have not been compared. In this study, we comprehensively compared the transcriptional profiling of IFN-λ3- and IFN-α-induced genes in a porcine intestinal epithelial cell line (IPEC-J2) and verified the RNA-Seq results by reverse transcriptase quantitative PCR (RT-qPCR) *in vitro*, and further confirmed the transcriptional profile difference in crypt-derived porcine enteroids.

## Materials and Methods

### Cells and Viruses

The intestinal porcine epithelial cell line J2 (IPEC-J2; kindly provided by Dr. Anthony Blikslager, North Carolina State University, Raleigh, NC, USA) was maintained in Dulbecco's Modified Eagle's Medium Nutrient Mixture F-12 (DMEM/F12) supplemented with antibiotics (100 units/ml penicillin and 100 μg/ml streptomycin), 0.1 mM HEPES (Gibco, USA), and 10% heat-inactivated fetal bovine serum (FBS) (Gibco). African green monkey kidney cells (Vero E6) were grown and maintained in DMEM supplemented with antibiotics (100 units/ml penicillin and 100 μg/ml streptomycin) and 10% heat-inactivated FBS (Gibco). PEDV strain CV777 of genotype 1 (GenBank Accession No. KT323979) was maintained at the Harbin Veterinary Research Institute of the Chinese Academy of Agricultural Sciences, Harbin.

### Determination of Antiviral Units (AUs) of IFN-λ3

The biological antiviral activity of *E. coli*-derived recombinant porcine IFN-lambda 3 was prepared in our laboratory and evaluated in MDBK cells using a recombinant vesicular stomatitis virus (VSV) with a GFP reporter as described previously (22, 24). The weight-activity unit (U/ml) of samples was calculated using porcine prokaryotic-derived IFN-α (4.0 × 10^8^ U/mg) (Prosit Sole Biotechnology, Co., Ltd., Beijing, China) as a reference.

### Porcine Intestinal Enteroid Culture and Media

Porcine intestinal crypts were prepared from specific pathogen-free piglets using previously described protocols (21). In brief, the intestine was flushed with cold PBS with antibiotics (100 units/ml penicillin and 100 μg/ml streptomycin), cut into 2-mm segments, and washed with cold PBS with antibiotics until the supernatant was clear. The washed intestinal pieces were suspended in 15 ml of Gentle Cell Dissociation Reagent (STEMCELL, Canada) and shaken at 100 rpm for 25 min to disassociate the crypts at room temperature (RT). The pellets of the intestinal pieces were suspended in 10 ml of cold PBS with 0.1% bovine serum albumin (BSA) and antibiotics (Pen-Strep) and passed through a 70-μm cell mesh. The crypt pellets were harvested by centrifugation at 200 × *g* at 4°C for 5 min and resuspended in 10 ml of cold DMEM/F12. After counting, the intestine crypts were resuspended in 25 μl of IntestiCult Organoid Growth Medium (STEMCELL, Canada) and 25 μl of Matrigel (BD Biosciences, USA) per 50 crypts and seeded into a 48-well plate at 50 crypts per well. The plate was incubated at 37°C for 10 min until the Matrigel solidified. The plate was filled with Complete IntestiCult Organoid Growth Medium and then incubated at 37°C in a 5% CO_2_ incubator. The culture medium was exchanged every 3–4 days. The Institutional Animal Care and Use Committee of the Harbin Veterinary Research Institute approved all the protocols related to the animal experiments performed in this study.

### Two-Dimensional (2D) Monolayer Enteroid Culture

Expanded 3D enteroids were recovered from the Matrigel after 7–11 days of growth by the addition of ice-cold DMEM/F12 medium, transferred into 15-ml tubes, and centrifuged at 250 × *g* at 4°C for 5 min. The pellet of enteroids was incubated in 0.25% Trypsin (Gibco) for 5 min at 37°C and dissociated by repeated pipetting to obtain a single-cell suspension. DMEM-F12 with 10% (v/v) FBS was added into the single-cell suspension, and the mixture was centrifuged at 800 × *g* for 5 min. The cell pellets were resuspended in complete IntestiCult Organoid Growth Medium at RT and seeded at 50 enteroids per well in a Matrigel-precoated 96-well plate. After differentiation for about 3–4 days, planar monolayers of 2D enteroids were ready for use in experiments.

### RNA Isolation and RT-qPCR

Total cellular RNA was extracted using the Simply P Total RNA Extraction Kit (BioFlux, China) according to the manufacturer's instructions. Total RNA (1 μg) was reverse-transcribed to cDNA using the PrimeScript™ II First-Strand cDNA Synthesis Kit (Takara, China). The synthesized cDNA was subjected to qPCR performed in triplicate using a LightCycler^®^ 480 II real-time PCR instrument (Roche, Switzerland) and SYBR Green PCR mix (Life Technologies, USA) according to the manufacturer's instructions. All the data were acquired and analyzed using LightCycler^®^ 480 II software 1.5 based on the cycle threshold (^ΔΔ^CT) method (25). GAPDH served as the internal control. The amplification efficiency of qPCR primers ranged from 85.83 to 106.38%. The primers used in this assay were designed using Primer Premier 5 software and are listed in Table 1.

**Table 1:** Primer sequences for PCR cloning and qPCR.

**Gene name**		**Primer sequences (5^**′**^-3^**′**^)**
qISG15	Forward	AGCATGGTCCTGTTGATGGTG
	Reverse	CAGAAATGGTCAGCTTGCACG
qOASL	Forward	TCCCTGGGAAGAATGTGCAG
	Reverse	CCCTGGCAAGAGCATAGTGT
qOAS1	Forward	GAGTTTTCCACCTGCTTCACG
	Reverse	AAATCTGTTTTCCCGCTTCCT
PEDV_CV777 ORF3	Forward	GCACTTATTGGCAGGCTTTGT
	Reverse	CCATTGAGAAAAGAAAGTGTCGTAG
qIFIT3	Forward	GCACCAAATTCATGGTATCTCC
	Reverse	TTCCTTCCTGTCTCTGTCAGCC
qGAPDH	Forward	CCTTCCGTGTCCCTACTGCCAAC
	Reverse	GACGCCTGCTTCACCACCTTCT
qISG12A	Forward	ACAGGGAGTCTGGCCAAAGCA
	Reverse	CCATCAAAGTCACAGGAGGGG
qPLAC8	Forward	TTTGTGATGACTTTATGGTG
	Reverse	GAGAAGCTGAAGAGGTGTGT
qIFIT2	Forward	TGAAATGTGTGGGAAAAGAGA
	Reverse	CAGAGGCAGGCGAGATAGGAG
qRSAD2	Forward	AAGCAGAGCAGTTTGTTATCAGC
	Reverse	TTCCGCCCGTTTCTACAGT
RSAD2	Forward	CCGCTCGAGATGTGGACACTGGTACCTG
	Reverse	GGAAGATCTTCACCAGTCCAGCTTCAGGTCC
qMX2	Forward	GGTGGACCCCGAAGGAGACAG
	Reverse	AAGTGCGGATGCGAGTGAAAG
MX2	Forward	GGGAATTCATGCCTAAACCCCGCATGTC
	Reverse	GGGGTACCTTACATCCCTTGTACCTCAAC

### RNA-Seq Analysis

Three biological replicates of each of the three groups, namely, untreated IPEC-J2 cells (mock control), IPEC-J2 cells treated with IFN-λ3 (1,000 ng/ml) for 24 h, or IPEC-J2 cells treated with IFN-α (1,000 ng/ml) for 24 h, were prepared for RNA sequencing. Total RNA was purified using the TRIzol reagent according to the manufacturer's instructions (Thermo Fisher Scientific, USA). The total RNA from each sample was quantified and qualified using an Agilent 2100 Bioanalyzer (Agilent Technologies, USA), a NanoDrop instrument (Thermo Fisher Scientific, Inc.), and a 1% agarose gel. One microgram of total RNA with a RIN value >7 was used for subsequent library construction. Next-generation sequencing library preparations were constructed according to the manufacturer's recommended protocol (NEBNext^®^ Ultra™ RNA Library Prep Kit for Illumina^®^). Poly mRNA isolation was performed using a NEBNext Poly(A) mRNA Magnetic Isolation Module (NEB) or Ribo-Zero™ rRNA Removal Kit (Illumina). mRNA fragmentation and priming were performed using NEBNext First-Strand Synthesis Reaction Buffer and NEBNext Random Primers. First-strand cDNA was synthesized using ProtoScript II Reverse Transcriptase, and second-strand cDNA was synthesized using a Second-Strand Synthesis Enzyme Mix. The purified double-stranded cDNA (by AxyPrep Mag PCR Clean-up kit, Axygen) was then treated with End Prep Enzyme Mix to repair both ends and add a dA tail through one reaction, and the product was subjected to T–A ligation to add adaptors to both ends. Size selection of adaptor-ligated DNA was then performed using an AxyPrep Mag PCR Clean-up kit (Axygen), and fragments of ~360 bp (with an approximate insert size of 300 bp) were recovered. Each sample was then amplified by PCR for 11 cycles using the P5 and P7 primers carrying sequences that can anneal during bridge PCR with a flowcell and the P7 primer carrying a six-base index to allow multiplexing. The PCR products were cleaned up using the AxyPrep Mag PCR Clean-up kit (Axygen), validated using an Agilent 2100 Bioanalyzer (Agilent Technologies, USA), and quantified with a Qubit 2.0 Fluorometer (Invitrogen, USA). Libraries with different indices were then multiplexed and loaded on an Illumina HiSeq instrument (Illumina, USA) according to the manufacturer's instructions. Sequencing was performed using a 2 × 150-bp paired-end (PE) configuration and image analysis and base calling were conducted using HiSeq Control Software (HCS) + OLB + GAPipeline-1.6 (Illumina) provided with the HiSeq instrument. RNA-Seq was performed and analyzed using GENEWIZ9 (Suzhou, China).

### Microarray Analysis

The analysis of the microarray data to identify differentially expressed genes was performed using edgeR software. The analysis starts with the count, and the data are standardized by TMM and then subjected to differential expression analysis. We selected | log_2_ (fold change) (logFC) | > 1 and FDR < 0.05 as the screening criteria for the identification of differentially expressed genes.

### Immunofluorescence Assay (IFA)

IPEC-J2 or Vero E6 cells were fixed with 4% paraformaldehyde for 30 min at RT. The fixed cells were permeabilized with 0.2% Triton X-100 for 20 min at RT and then blocked with blocking buffer (PBS with 5% FBS and 5% skim milk) for 30 min at 37°C. The cells were then incubated with PEDV N protein antibody at 37°C for 2 h and then labeled with Alexa Fluor 546 goat anti-mouse IgG antibody (Thermo Fisher Scientific, USA) at 37°C for 1 h. DAPI (Sigma, USA) was used for the staining of cellular nuclei. The stained cells were visualized using an AMG EVOS F1 fluorescence microscope (Thermo Fisher Scientific, USA).

### Construction of Eukaryotic Expression Plasmids

The porcine RSAD2 or MX2 coding region was amplified by RT-PCR, and cDNA was prepared using the PrimeScript™ II 1st Strand cDNA Synthesis Kit (Takara, China). RSAD2 or MX2 was then amplified using a pair of primers specific for porcine RSAD2 or MX2 (Table 1), respectively. The PCR products were purified, digested, and cloned into pCAGGS-HA through *EcoR I* and *Kpn I*. Construction of the pRSAD2/pMX2-HA expression plasmid was confirmed by sequencing. Vero E6 cells were grown in 48-well plates to 70–80% confluence and then transfected with the pRSAD2/pMX2-HA expression plasmid using the Attractene Transfection Reagent (Qiagen), and the expression of pRSAD2/pMX2 was verified by anti-HA IFA.

### Statistical Analyses

GraphPad Prism software version 7 was used to analyze the experimental results. The results are expressed as scatter plots in which the mean is indicated by a line. The differences between groups were compared using an unpaired Mann–Whitney test. *P* < 0.05 was considered significant, and the *p-*values are expressed as follows: ^*^p < 0.05, ^**^p < 0.01, ^***^p < 0.005, and ^****^p < 0.001.

## Results

### The Inhibition of PEDV in IPEC-J2 Cells by Exogenous IFN-λ3 Is Superior to That Achieved With IFN-α

**Figure 1:**
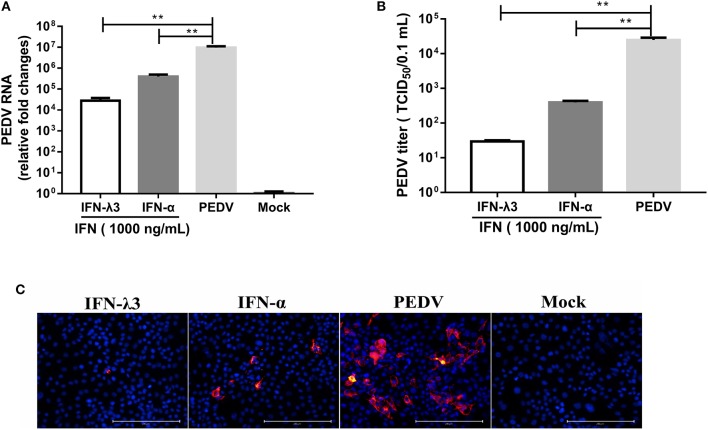
IFN-λ3 inhibits PEDV infection and is superior to IFN-α in IPEC-J2 cells. **(A,B)** Inhibition of PEDV infection in IPEC-J2 cells by IFN-λ3 or IFN-α. **(A)** IPEC-J2 cells were primed with IFN (1,000 ng/ml) for 24 h and infected with PEDV CV777 at an MOI of 1, and the PEDV viral genome numbers **(A)** and titers **(B)** at 48 hpi were determined by RT-qPCR and TCID_50_ titration, respectively. The results are presented as the means ± SEMs (*n* = 3). The differences between groups were compared using an unpaired Mann-Whitney test. *P* < 0.05 was considered significant, and the *p* values are expressed as follows: ^*^p < 0.05, ^**^p < 0.01, ^***^p < 0.005, and ^****^p < 0.001. **(C)** Representative images of inhibition of PEDV infection in IPEC-J2 cells by IFN-λ3 or IFN-α. IPEC-J2 cells were primed with IFN (1,000 ng/ml) for 24 h and infected with PEDV CV777 at an MOI of 1, and the cells were fixed with 4% paraformaldehyde at 48 hpi, and the expression of PEDV N protein was detected with mouse anti-PEDV N mAb (red). DAPI was used to stain cellular nuclei (blue). Bar = 200 μm.

According to our study and other previous studies (21–23), IFN-λ1, IFN-λ3, and IFN-α all significantly inhibit PEDV infection, but IFN-λ3 shows the strongest antiviral activity against PEDV. To confirm this disparity in the antiviral activities of IFN-λ3 and IFN-α against PEDV, IPEC-J2 cells were primed with IFN-α (1,000 ng/ml) or IFN-λ3 (1,000 ng/ml) for 24 h and then infected with PEDV (MOI = 1). Consistent with our previous results, both IFN-α and IFN-λ3 substantially suppressed PEDV infection in IPEC-J2 cells, as demonstrated by measurements of the viral genomes and titers by RT-qPCR (Figure 1A) and TCID_50_ titration (Figure 1B), respectively. IFN-α reduced the number of PEDV genomes by 24-fold, whereas IFN-λ3 decreased the number of PEDV genomes by approximately 342-fold. The virus titers were consistent with results of PEDV genomes: PEDV titers decreased 10- and 100-fold after pretreatment with IFN-α or IFN-λ3, respectively. The inhibition of PEDV infection by both IFN-α and IFN-λ3 was further verified by PEDV N protein IFA (Figure 1C). The virus-infected cells were significantly decreased after pretreatment with IFN-α or IFN-λ3, whereas the number of PEDV-infected cells in the IFN-λ3-pretreated group was significantly decreased, as demonstrated by only a few sporadically distributed cells, compared with those in the IFN-α-pretreated group. Thus, IFN-λ3 restricted PEDV infection in IPEC-J2 cells more effectively than IFN-α, regardless of the quantification of viral RNA, infectivity, or viral protein (Figure 1).

### IFN-λ3 Induces a Unique Transcriptional Profile in IPEC-J2 Cells Compared With That Induced by IFN-α

**Figure 2:**
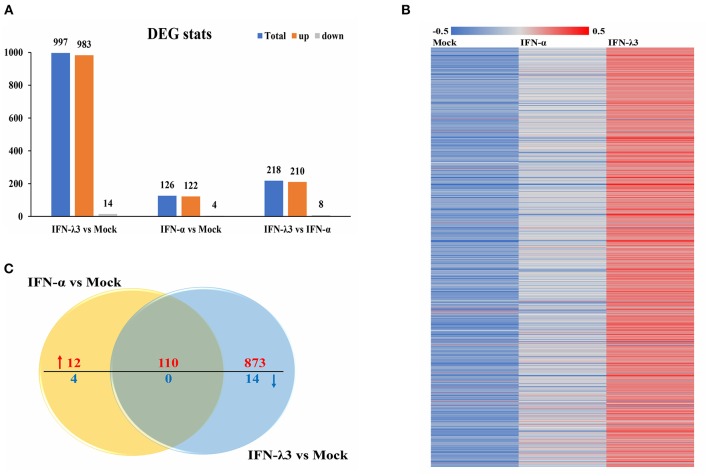
Comparative transcriptome analysis of IFN-λ3- or IFN-α-treated IPEC-J2 cells. **(A)** The graph of statistical results of IFN-λ3 or IFN-α differential expression. IPEC-J2 cells were primed or not primed with IFN-λ3 (1,000 ng/ml) and IFN-α (1,000 ng/ml) for 24 h. Then, the total RNA isolated from the cells was used for RNA sequencing. **(B)** The heat map of gene expressed induced by IFN-λ3 or IFN-α. **(C)** The venn map analysis of common differentially expressed genes elicited by IFN-λ3 or IFN-α.

To assess the underlying mechanisms of the disparity between the anti-PEDV responses induced by IFN-α or IFN-λ3, we performed an RNA-sequencing (RNA-Seq) analysis of total cellular RNA isolated from IPEC-J2 cell cultures stimulated with IFN-α or IFN-λ3 for 24 h and IFN untreated IPEC-J2 mock control. This duration of stimulation (24 h) was selected based on the efficacy of viral inhibition after exposure to IFN-λ3 and IFN-α (Figure 1) (22). Each of the triple replicate yielded more than 3.89 × 10^7^ clean reads and has more than 95% Q20 (%), indicating the reliability of the RNA-Seq data. The IFN-λ3-stimulated cells showed a total of 997 differentially expressed genes, including 983 upregulated genes and 14 downregulated genes, compared with the mock control, whereas IFN-α only upregulated the expression of 122 genes among the total of 126 differentially expressed genes and reduced the expression of four genes (Figure 2A). The number of IFN-λ3-modified genes was approximately 10-fold higher than that of IFN-α-modified genes. These results indicate that the intestine epithelia respond better to IFN-λ3 than to IFN-α. We further grouped the corresponding genes through supervised partitioning clustering and combined the differentially expressed genes induced by IFN-λ3 and IFN-α to obtain the heat map (Figure 2B) and the venn map (Figure 2C). IFN-λ3 yielded different gene expression profiles compared with that induced by IFN-α, even though these showed substantial overlap (Figure 2B). One hundred ten genes were upregulated in both the IFN-λ3- and IFN-α-treated cells, whereas 873 and 12 genes were uniquely upregulated in the presence of IFN-λ3 and IFN-α, respectively (Figure 2C). None of the coexpression genes were downregulated in both the IFN-λ3- and IFN-α-treated cells, whereas 14 and 4 genes were only downregulated in the presence of IFN-λ3 and IFN-α, respectively. These results demonstrated that IFN-λ3 induces a unique gene transcriptional profile in the intestine epithelia compared with IFN-α.

### IFN-λ3 Exerts a Stronger Effect on the Biological Function of Intestinal Epithelial Cells Than IFN-α

**Figure 3:**
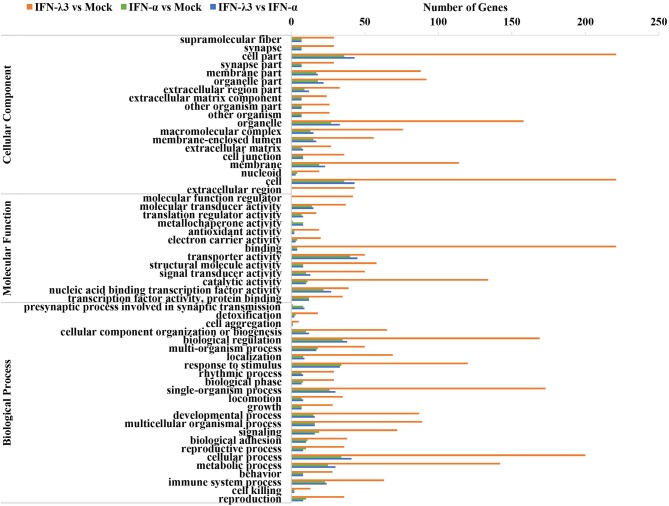
GO enrichment analysis of IFN-λ3 or IFN-α targets.

To further clarify the functional consequences of the gene profiles elicited by either IFN-λ3 or IFN-α, we performed a gene ontology (GO) enrichment analysis using a database established by the Gene Ontology Consortium, which aims to define and describe the functions of genes and proteins in various species (Figure 3) (26). Among the 983 genes upregulated by IFN-λ3, 221 and 134 genes (22.17 and 13.44% of the total genes, respectively) were associated with a binding function and catalysis, respectively, and these two functions account for the main molecular functional changes. The dominant functions of the IFN-α-regulated genes are cellular transporter activity and nucleic acid-binding transcription factor activity, and these functions were associated with 40 genes (31.75% of total genes) and 22 genes (17.46% of total genes), respectively. The analysis of cellular components revealed that 221 differentially expressed genes in the IFN-λ3-treated group affected the cell part, and 36 genes associated with this cellular component were differentially expressed in the IFN-α-treated group. With respect to biological processes, the IFN-λ3-treated group included 200, 173, 169, 142, and 120 differentially expressed genes associated with the cellular process, the single-organism process, biological regulation, the metabolic process, and response to stimulus, respectively. The pattern obtained for the IFN-α-treated group was similar to that of the IFN-λ3-treated group, and the differentially expressed genes were also concentrated on the following five functions: the cellular process, the single-organism process, the biological regulation, the metabolic process, and the response to stimulus. However, the numbers of differentially expressed genes after IFN-α treatment were notably lower than those obtained after IFN-λ3 treatment. These results indicated that both IFN-λ3 and IFN-α are involved in the regulation of multiple cellular processes in IPEC-J2 cells, such as cellular components and molecular functions, but IFN-λ3 exerts more potent effects than IFN-α. To further investigate the function of genes specifically induced by IFN-λ3, we extracted the transcriptional profiles of IFN-λ3 and IFN-α regarding biological processes (*p* < 0.01) and combined them based on the –log_10_(*p*) values to obtain a heat map that showed the enrichment of biological processes (Figure S1). The data demonstrated that in IPEC-J2 cells, IFN-λ3 stimulation triggered more biological reaction processes than IFN-α, and these processes mainly involved the modulation of metabolic processes, including cellular metabolic processes, macromolecular metabolic processes, and primary metabolic regulation. Taken together, these results indicate that the differentially expressed genes induced by IFN-λ3 are involved in more intracellular biological processes than those induced by IFN-α.

### KEGG Pathway Enrichment and Interaction Analysis of Differentially Expressed Genes

**Figure 4:**
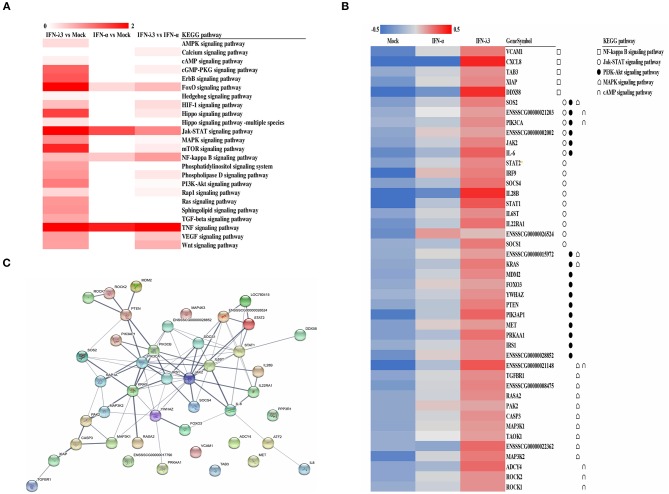
KEGG pathway enrichment and interaction analysis of differentially expressed genes induced by IFN-λ3 or IFN-α. **(A)** KEGG pathway enrichment analysis of differential genes induced by IFN-λ3 or IFN-α. The differential genes induced by IFN-λ3 or IFN-α were analyzed using software kobas3.0. **(B)** The expression heat map of differential expression genes induced by IFN-λ3 or IFN-α. The RPKM values of differentially expressed genes of IFN-λ3 or IFN-α were extracted, and the same set of different replicates was averaged. The average of the RPKM values of all samples of a certain gene was calculated, and the heat map was made by log 10 (the average RPKM value of a certain gene of each group/the average RPKM value of the gene). The KEGG pathway to which the gene belongs is indicated by a different symbol. **(C)** Protein interaction prediction analysis of differentially expressed genes by string database. The relevant genes were input into string (http://www.string-db.org/), the active interaction sources were selected as homology, and the experimentally determined interaction and database annotated were used as predictive conditions for protein interaction prediction. The thickness of the line represents combined score, which is the combined score of the prediction results of the three prediction conditions, indicating the strength of data support.

To explore the clustering of the IFN-induced differential expression genes in anti-viral signaling pathways, we performed a KEGG enrichment analysis of the differentially expressed genes using KOBAS 3.0. The KEGG enrichment analysis revealed that the differentially expressed genes induced by IFN-λ3 involved in much broader signal pathways compared with IFN-α though they both primarily modified the NF-κB signaling pathway, the Jak-STAT signaling pathway, the phosphoinositide-3-kinase-Akt (PI3K-Akt) signaling pathway, the mitogen-activated protein kinase (MAPK) signaling pathway, and the cGMP-PKG signaling pathway (Figure 4A). To determine the signaling pathways involving the differentially expressed genes induced by IFN-λ3 or IFN-α, we combined the differentially expressed genes and analyzed their expression patterns (Figure 4B). The results showed that the differentially expressed genes were most abundantly involved in the Jak-STAT signaling pathway, followed by the PI3K-Akt signaling pathway and the MAPK signaling pathway, which is consistent with the finding that the Jak-STAT signaling pathway primarily mediates IFN-induced antiviral responses. Among the proteins encoded by the differentially expressed genes, SOS2, PIK3CA, JAK2, IL-6, SOCS4, IL-28B, STAT1, IL22RA1, and SOCS1 play a major role in the Jak-STAT signaling pathway, and KRAS, PIK3AP1, PRKAA1, ENSSSCG00000021148, ENSSSCG00000022362, and MAP3K2 play a major role in the PI3K-Akt signaling pathway. To predict the protein interactions between differentially expressed genes, we extracted the union of differentially expressed genes and introduced these into the web-based tool STRING (http://www.string-db.org/) to generate protein–protein interaction networks (Figure 4C). The results of the STRING analysis indicated that JAK2, STAT2, PTEN, PIK3CA, IRS1, KRAS, and IL6ST are closely related to other proteins and displayed significant differential expression compared with the IFN-α-induced proteins, which play critical roles in the innate immune response induced by IFN. Collectively, the results showed that the IFN-λ3-induced differentially expressed genes are involved in more signaling pathways, particularly those associated with innate immunity, than those induced by IFN-α, which indicates that IFN-λ3 exhibits stronger antiviral activity in intestinal epithelial cells.

### IFN-λ3 Induces Specific Antiviral Gene Expression Compared With IFN-α

**Figure 5:**
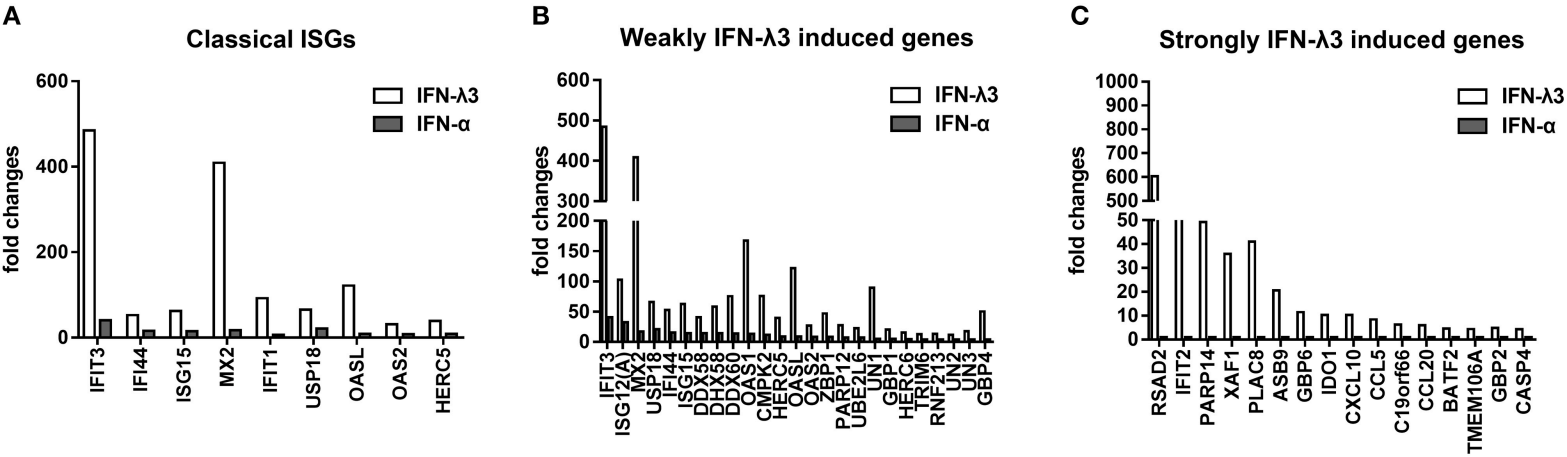
Graphical representation showing the fold change analysis of different gene subgroups [“classical antiviral IFN-stimulated genes (ISGs),” “weakly IFN-λ3-induced genes,” and “strongly IFN-λ3-induced genes”]. **(A)** The classical antiviral IFN-stimulated gene (ISG) subgroup. “Classical ISGs” were defined by their designation in the literature. **(B)** The weakly IFN-λ3-induced gene subgroup. “Weakly IFN-λ3-induced genes:” fold increase by IFN-λ3 over mock control/fold increase by IFN-α over mock control >2 and fold increase by IFN-α over mock control >4. Unknown genes were named as UN1, UN2, and UN3. **(C)** The strongly IFN-λ3-induced gene subgroup. “Strongly IFN-λ3-induced genes:” fold increase by IFN-λ3 over mock control/fold increase by IFN-α over mock control >4 and fold increase by IFN-α over mock control <2.

To further elucidate the mechanisms underlying the antiviral activity discrepancy between IFN-λ3 and IFN-α, we focused on the top 100 IFN-λ3-induced genes (fold change compared with the mock control) and divided them into three subgroups (classical ISGs, weakly IFN-λ3-induced genes, and strongly IFN-λ3-induced genes) as in a previous study (16). The expression of the 100 top genes in all three subgroups induced by IFN-λ3 was significantly higher compared with that induced by IFN-α (Figure 5). The ISGs in the classic ISG subgroup are primarily the classical ISGs induced by IFN reported in the literature (16, 27). The levels of ISGs in this subgroup induced by IFN-λ3 were from 3- to 23-fold higher than those induced by IFN-α. The weakly IFN-λ3-induced gene subgroup contained 25 genes, including three unknown genes (being denoted UN1, UN2, and UN3). The analysis of this subgroup showed that both IFN-λ3 and IFN-α induced a more than 4-fold increase in the expression of ISGs compared with mock control. The IFN-λ3-induced expression levels of IFIT3, OASL, OAS1, and GBP4, which are important innate immunity factors, were 10-fold higher than those induced by IFN-α. The expression of 16 of the genes in the strongly IFN-λ3-induced gene subgroup was strongly induced by IFN-λ3, whereas IFN-α did not induce a substantial increase in expression (<2-fold compared with the mock control). Interestingly, radical S-adenosyl methionine domain containing 2 (RSAD2), a multifunctional protein with broad antiviral activity that can inhibit both DNA and RNA viruses, is the top gene upregulated by IFN-λ3 (28). In contrast, IFN-α did not substantially upregulate RSAD2 expression. Several other genes (IFIT2, PARP14, and GBP6) in this subgroup are also important innate viral restriction factors (27, 29–31). Thus, IFN-λ3 induces a stronger antiviral innate immune response compared with IFN-α.

### RT-qPCR Verification of the Disparity in the Transcription Profiles Induced by IFN-λ3 or IFN-α in IPEC-J2 Cells Identified by RNA-Seq

**Figure 6:**
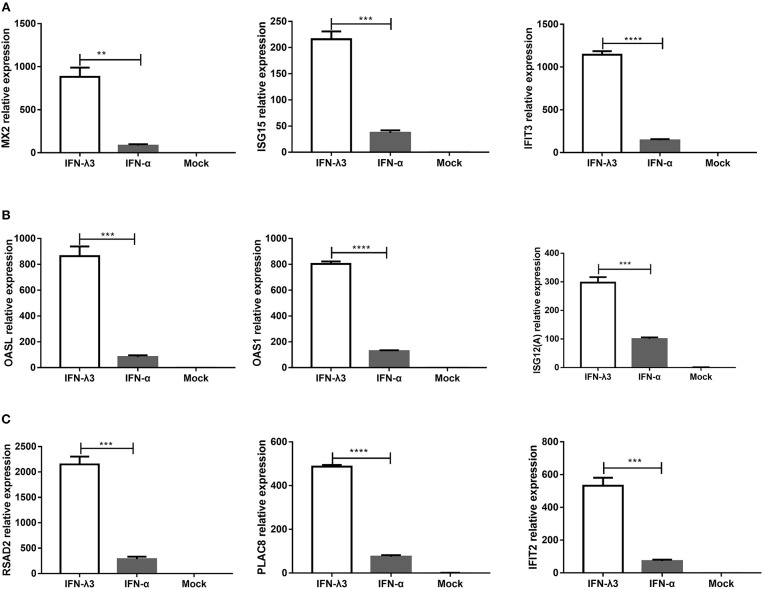
Confirmation of differential expression profiles of three IFN-λ3-induced gene subgroups induced by IFN-λ3 and IFN-α in IPEC-J2 cells. **(A–C)** Validation of **(A)** classical ISGs subgroup, **(B)** weakly IFN-λ3-induced genes subgroup, and **(C)** strongly IFN-λ3-induced genes subgroup by RT-qPCR. The differences between groups were compared using an unpaired Mann-Whitney test. *P* < 0.05 was considered significant, and the *p* values are expressed as follows: ^*^p < 0.05, ^**^p < 0.01, ^***^p < 0.005, and ^****^p < 0.001.

To verify the unique antiviral gene expression profile induced by IFN-λ3 that was detected by RNA-Seq, as shown in Figure 5, we randomly selected three genes from each group to be verified by RT-qPCR. The analysis of the three subgroups confirmed that IFN-λ3 most substantially upregulated the expression of ISGs (Figure 6). Specifically, IFN-λ3 upregulated the expression of large distinct classes of ISGs in the classical ISG subgroup, such as MX2, ISG15, and IFIT3, the ISG fold changes induced by IFN-λ3 were up to nearly 10-fold higher than those induced by IFN-α (Figure 6A). The analysis of the weakly IFN-λ3-induced gene subgroup showed that the expression of OASL, OAS1, and ISG12 (A) was induced by both IFN-λ3 and IFN-α to significantly different levels. The OASL and OAS1 fold changes induced by IFN-λ3 were up to 8-fold greater than those induced by IFN-α (Figure 6B). More substantial fold changes in the expression of ISGs in the strongly IFN-λ3-induced gene subgroup were obtained with IFN-λ3 compared with IFN-α, which resulted in only slight changes in expression (Figure 6C). RSAD2, PLAC8, and IFIT2 were more substantially upregulated by IFN-λ3 than by IFN-α. Collectively, the *in vitro* RT-qPCR results confirmed that IFN-λ3 induces stronger ISG expression compared with IFN-α.

### The Different Transcription Profiles in Enteroids Induced by IFN-λ3 or IFN-α

**Figure 7:**
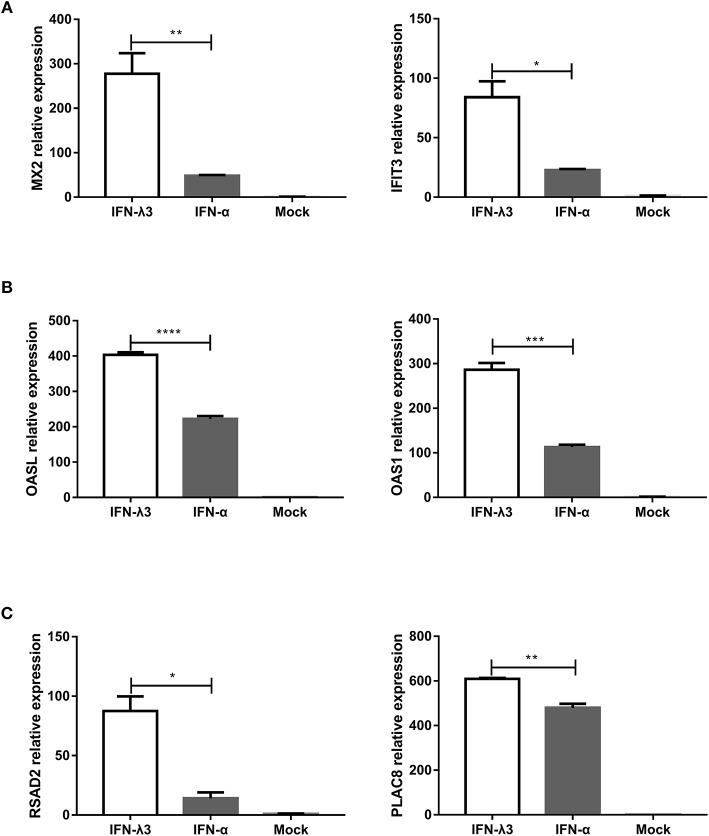
Verification of expression profiles of differentially expressed genes induced by IFN-λ3 or IFN-α in porcine enteroids by RT-qPCR. **(A–C)** Validation of **(A)** the classical ISG subgroup, **(B)** the subgroup of weakly IFN-λ3-induced genes, and **(C)** the subgroup of strongly IFN-λ3-induced gene by RT-qPCR. Enteroids were or were not primed with IFN-λ3 (1,000 ng/ml) or IFN-α (1,000 ng/ml) for 24 h, and the total RNA from the enteroids was extracted and used for RT-qPCR. The differences between groups were compared using an unpaired Mann-Whitney test. *P* < 0.05 was considered significant, and the *p* values are expressed as follows: ^*^p < 0.05, ^**^p < 0.01, ^***^p < 0.005, and ^****^p < 0.001.

Enteroids derived from intestinal crypt stem cells, which mimic the diverse cellular nature and physiological activity of the small intestine while also maintaining the genetic identity of the host, constitute a unique *ex vivo* model for studying the intestine (21, 27). To further confirm whether the IFN-λ3-induced gene expression profile in enteroids was the same as that in IPEC-J2 cells, we stimulated enteroids with IFN-λ3 or IFN-α under the same conditions as IPEC-J2 cells and evaluated their gene expression by RT-qPCR. In all three groups, the expression pattern of the genes induced by IFN-λ3 and IFN-α in porcine enteroids was consistent with that found in the IPEC-J2 cells (Figure 7). In the classical ISG subgroup, the upregulated levels of MX2 and IFIT3 elicited by IFN-λ3 were ~3- to 5-fold higher than those induced by IFN-α (Figure 7A). In contrast, the upregulated levels of OASL and OAS1, which belong to the weakly IFN-λ3-induced gene subgroup, induced by IFN-λ3 were 2-fold higher than those induced by IFN-α, and the fold change difference was moderate (Figure 7B). For the strongly IFN-λ3-induced gene RSAD2 and PLAC8, just as we observed in the other subgroups, IFN-λ3 significantly induced higher expression than IFN-α does (Figure 7C). In summary, IFN-λ3 induces higher expression of ISGs than IFN-α in porcine enteroids, which suggests that IFN-λ3 exerts a greater effect in gastrointestinal epithelial cells than IFN-α.

### pMX2 and pRSAD2 Inhibit PEDV Infection in Vero E6 Cells

**Figure 8:**
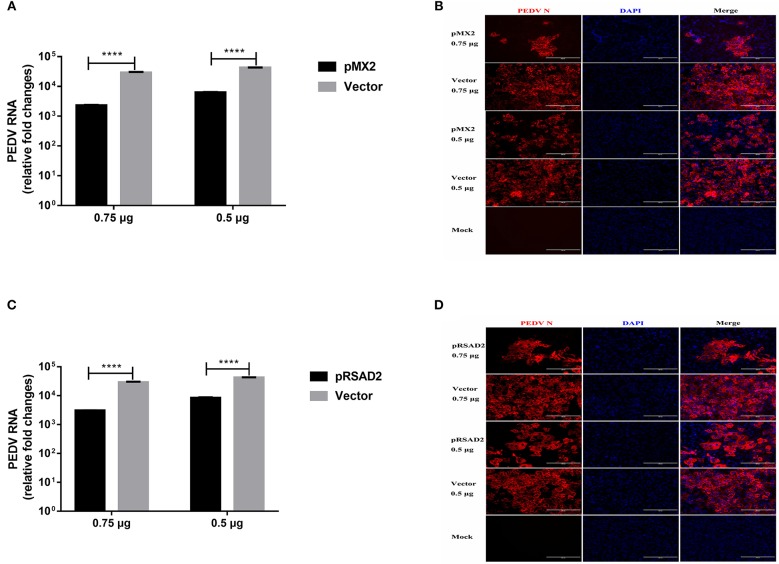
Inhibition of PEDV infection by IFN-λ3 selectively elicited pMX2 or pRSAD2. **(A–D)** The overexpression of pMX2 or pRSAD2 inhibited PEDV infection. pMX2 or pRSAD2 was cloned and expressed in the eukaryotic expression vector pCAGGS-HA. Vero E6 cells were transfected with pMX2 **(A,B)** or pRSAD2 **(C,D)** and pCAGGS-HA for 24 h and then were infected with PEDV CV777 (MOI = 1). PEDV infection was determined by measuring PEDV genomes **(A,C)** and PEDV N protein **(B,D)**. The differences between groups were compared using an unpaired Mann-Whitney test. *P* < 0.05 was considered significant, and the *p* values are expressed as follows: ^*^p < 0.05, ^**^p < 0.01, ^***^p < 0.005, and ^****^p < 0.001.

We selected MX2 (classical ISG) and RSAD2 (strongly IFN-λ3-induced gene) among the top 10 genes induced by IFN-λ3 to evaluate the antiviral effect of the IFN-λ3-induced expression of ISGs against PEDV infection. We cloned porcine *mx2* or *rsad2* into the eukaryotic expression vector pCAGGS-HA, and the transient expression of pMX2 or pRSAD2 in Vero E6 cells was verified by IFA (data not shown). As expected, pMX2 or pRSAD2 transient overexpression significantly inhibited PEDV infection in Vero E6 cells, as demonstrated by measuring the viral RNA (Figures 8A,C) and PEDV N protein expression by IFA (Figures 8B,D). Thus, these data indicate that the differentially upregulated pMX2 or pRSAD2 serves as an important IFN-λ3-elicited antiviral host factor against PEDV.

## Discussion

Type I and type III IFNs, which establish a cellular antiviral state and restrict viral infection in the host, are key players at the earliest stages of innate immunity against viral infection. Despite the similarities between the effects of the two types of IFNs, increasing evidence demonstrates that each class of IFN is essential for antiviral host defense and is not functionally redundant (2). A previous study in mice demonstrated that the role of IFN-λ in functionally redundant intestinal viral infections cannot be compensated by IFN-α/β (20). Therefore, clarification of the induction of cell-specific IFN signaling profiles is essential for understanding the non-redundant roles of IFN-λ and IFN-α in viral infection at the tissue and organism levels. In this study, we found that the transcriptional profile induced by type III IFN in the intestinal epithelia was unique compared with that induced by type I IFN. Compared with IFN-α, stimulation with IFN-λ3 not only resulted in higher levels of most ISGs but also substantially increased diversity in the gene profiles involved in various cellular functions.

Type I and III IFNs each signal through different heterodimeric complex receptors to initiate multiple downstream signaling pathways. In addition to activation of the Jak-STAT signaling pathway, IFNs also activate the PI3K and MAPK signaling cascades (5, 32). The combined use of these signaling pathways by IFNs and many other cytokines might help explain the different roles of IFNs in regulating the antiviral and immune responses in a variety of environments and locations. We have very limited knowledge on the pathways activated by IFN-λ, which are important for understanding the immune-modulating activities of IFN-λ (8). In this study, we found that IFN-λ3 not only activates the classical antiviral response Jak-STAT signaling pathway but also primarily activated the NF-κB signaling pathway, the cAMP signal pathway, the PI3K-Akt signaling pathway, and MAPK signaling pathway (Figure 4). IFN-λ3 activated much more signaling pathways in epithelia than IFN-α, and this finding might provide new insights into the mechanism through which IFN-λ modulates other cellular functions beyond its direct antiviral activity. IFN-λ3 largely stimulated Wnt signaling pathway in IEC compared with IFN-α, which plays critical roles in maintaining the homeostasis of intestinal epithelia *in vivo* (33). Further studies of different signaling pathways induced by IFN-λ3 or IFN-α will help dissect the complex interaction of IFN-induced signaling pathways with similar or overlapping intracellular signaling pathways stimulated by other cytokines.

Most somatic cells can induce and respond to type I IFNs, but certain specialized cells, such as those in the mucosal epithelia, selectively produce and respond to type III IFNs during various virus infections. We demonstrated that the porcine intestinal epithelia respond to both type I and III IFNs, even though these IFNs induced different ISG expression levels. Type III IFNs comprise four functional and highly homologous subtypes, namely, IFN-λ1, IFN-λ2, IFN-λ3, and IFN-λ4, in humans. Our study and other studies have revealed differences in antiviral activity among different IFN-λ subtypes (22, 23, 34). IFN-λ3 is superior or equal to IFN-λ1 in terms of its antiviral activity in the intestinal epithelia (22) and the immortalized liver cell line HepG2 *in vitro* (34). The different kinetics and magnitude of ISG induction after stimulation might account for the variation in the antiviral activity among different types or subtypes IFNs. Previous studies that attempted to identify the gene profiles induced by type III IFNs primarily focused on the comparison of IFN-λ1 with type I IFN (15), and most of these studies were performed in immortalized human or mouse cell lines. The gene transcription profile induced by IFN-λ3, particularly the gene transcription profile induced by IFN-λ3 in porcine intestinal epithelial cells, has not been reported. Here, we performed comparative analyses of the transcriptional profiles induced by IFN-λ3 and IFN-α in porcine intestinal epithelia cells. More importantly, in this study, we verified the RNA-Seq results in primary swine crypt-derived enteroids, an *in vitro* system that well recapitulates the complicated cellularity of the GI tract, which exhibits a potent response to both type I and III IFNs (21, 27, 35). The differential expression in IPEC-J2 or enteroids detected by qPCR is not related to the selection of housekeeping gene GAPDH since we observed the same pattern when using another housekeeping gene actin (data not shown). Therefore, studying the differences among IFN-λ3- and IFN-α-induced gene expression in enteroids is more realistic and clinically significant.

The different antiviral activities of IFN-λ3 and IFN-α are likely due to the different degrees of ISG induction by these two cytokines, and IFN-λ3 induced increased ISG expression. The functions of some of the genes that were strongly induced by IFN-λ3 have been reported, but the functions and mechanisms of more genes remain unclear. RSAD2, a host viral restrictor gene, showed the highest upregulated levels after IFN-λ3 rather than IFN-α stimulation, whereas MX1 was the most highly upregulated gene after IFN-α stimulation. A previous study demonstrated that porcine RSAD2 effectively inhibited CSFV replication *in vitro*, and this effect might occur via the interaction of RSAD2 with CSFV E2 protein in the cytoplasm (28). Our study found that RSAD2 transient expression also substantially curtailed PEDV infection in Vero E6 cells *in vitro*, but the mechanism is unclear. MX2, an IFN-induced GTP-binding protein (36, 37), reportedly inhibits the replication of lentivirus HIV-1, simian immunodeficiency virus (SIV), and equine infectious anemia virus (EIAV) *in vitro* (38, 39). The function of porcine MX2 has been poorly studied. We confirmed that porcine MX2 inhibits PEDV infection in Vero E6 cells. We also discovered some unknown genes that were differentially expressed in response to IFN-λ3 or IFN-α stimulation, and this finding might provide a foundation for further elucidation of the different mechanisms of action of IFN-λ3 and IFN-α.

In summary, we compared the transcriptional profiles of IFN-λ3 and IFN-α in IPEC-J2 cells and identified a unique set of genes that were strongly induced by IFN-λ3 compared with IFN-α. The transcriptional enrichment analysis indicated that IFN-λ3 or IFN-α is involved in the regulation of cellular processes, such as cellular components and molecular functions, in IPEC-J2 cells, and that IFN-λ3 activates more robust signaling pathways, particularly the antiviral Jak-STAT signaling pathway, compared with IFN-α. IFN-λ3 preferentially upregulates antiviral genes in epithelial cells compared with IFN-α. These results indicate that IFN-λ3 selectively targets small intestinal epithelial cells and induces a non-redundant antiviral host response at the enteric mucosal site.

## Data Availability Statement

All datasets generated in this study are included in the manuscript/Supplementary Material.

## Ethics Statement

The animal study was reviewed and approved by the Harbin Veterinary Research Institute of the Chinese Academy of Agricultural Sciences, Harbin. Written informed consent was obtained from the owners for the participation of their animals in this study.

## Author Contributions

PL, LL, and LF designed the research studies and analyzed and interpreted the data. LL, MX, FF, and LY conducted experiments and acquired data. LL and PL drafted the manuscript and all authors contributed revisions.

### Conflict of Interest

The authors declare that the research was conducted in the absence of any commercial or financial relationships that could be construed as a potential conflict of interest.

## Supplementary Material

Figure S1 The heat map of IFN-λ3 and IFN-α transcriptional profile biological process enrichment analysis.
