# Supplementary figures and images for: IFN-Lambda 3 Mediates Antiviral Protection Against Porcine Epidemic Diarrhea Virus by Inducing a Distinct Antiviral Transcript Profile in Porcine Intestinal Epithelia
Source: Front Immunol. 2019 Oct 17;10:2394. doi: 10.3389/fimmu.2019.02394 (PMC6811514; doi:10.3389/fimmu.2019.02394)

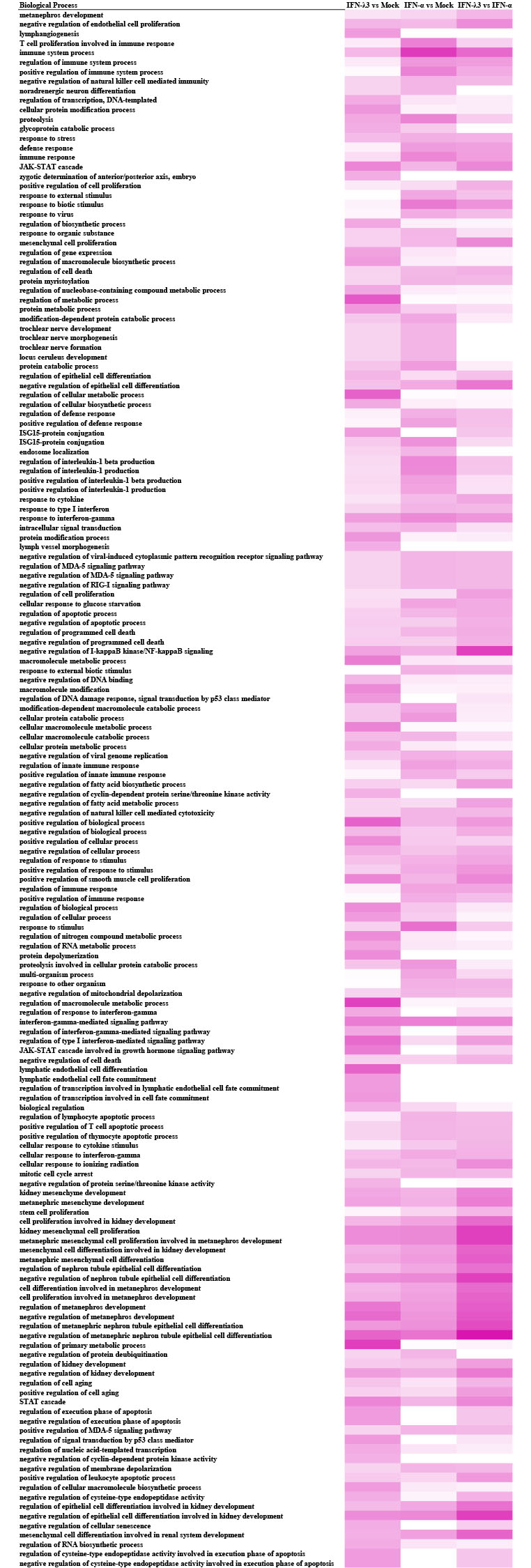

Supplement: Figure S1 — The heat map of IFN-λ3 and IFN-α transcriptional profile biological process enrichment analysis. [file Image_1.JPEG]
